# Quantitative assessment of HLA-DQ gene polymorphisms with the development of hepatitis B virus infection, clearance, liver cirrhosis, and hepatocellular carcinoma

**DOI:** 10.18632/oncotarget.22941

**Published:** 2017-12-05

**Authors:** Tao Xu, Anyou Zhu, Meiqun Sun, Jingzhu Lv, Zhongqing Qian, Xiaojing Wang, Ting Wang, Hongtao Wang

**Affiliations:** ^1^ Department of Clinical Laboratory, Bengbu Medical College, Bengbu, Anhui, P. R. China; ^2^ Clinical Testing and Diagnose Experimental Center of Bengbu Medical College, Bengbu, Anhui, P. R. China; ^3^ Department of Clinical Laboratory Science, The First Affiliated Hospital of Bengbu Medical College, Bengbu, Anhui, P. R. China; ^4^ Department of Histology and Embryology, Bengbu Medical College, Bengbu, Anhui, P. R. China; ^5^ Department of Biochemistry and Molecular Biology, Bengbu Medical College, Bengbu, Anhui, P. R. China; ^6^ Department of Immunology, Bengbu Medical College, Bengbu, Anhui, P. R. China; ^7^ Anhui Key Laboratory of Infection and Immunity, Bengbu Medical College, Bengbu, Anhui, P. R. China; ^8^ Anhui Clinical and Preclinical Key Laboratory of Respiratory Disease, The First Affiliated Hospital of Bengbu Medical College, Bengbu, Anhui, P. R. China; ^9^ Department of Internal Medicine, College of Medicine-Phoenix, The University of Arizona, Phoenix, AZ, USA

**Keywords:** HLA-DQ, Hepatitis B virus, Polymorphism, liver cirrhosis, hepatocellular carcinoma, Immunology

## Abstract

Hepatitis B is one of the most common infectious diseases, which leads to public health problems in the world, especially in Asian counties. In recent years, extensive human genetic association studies have been carried out to identify susceptible genes and genetic polymorphisms to understand the genetic contributions to the disease progression of HBV infection. *HLA-DQ* gene variations have been reported to be associated with HBV infection/clearance, disease progression and the development of hepatitis B-related complications, including liver cirrhosis (LC) and hepatocellular carcinoma (HCC). However, the results are either inconclusive or controversial. Therefore, to derive a more precise estimation of the association, a meta-analysis was performed. Our data revealed that the *HLA-DQ* alleles *rs2856718-G*, *rs7453920-A* and *rs9275319-G* were significantly associated with decreased risk of HBV infection and HBV natural clearance. Logistic regression analyses showed that *HLA-DQ* alleles *rs9275572-A* significantly increased HBV infection clearance, and decreased HBV natural clearance. However, *rs2856718-G* and *rs9275572-A* were not associated with development of cirrhosis. The *HLA-DQ* polymorphisms (*rs2856718* and *rs9275572*) were associated with a decreased HBV-related HCC risk in all genetic models, but *rs9272105-A* increased the risk of HBV-related HCC. In addition, no significant association was observed between *HLA-DQ rs9275319-G* polymorphism and HBV-related HCC. These stratified analyses were limited due to relatively modest size of correlational studies. In future, further investigation on a large population and different ethnicities are warranted. Our findings contribute to the personalized care and prognosis in hepatitis B.

## INTRODUCTION

Hepatitis B is an infectious disease caused by hepatitis B virus (HBV), which leads to the serious public health problems worldwide, especially in Asian counties. Nowadays, there are more than 240 million HBV carriers [1], among which 0.5-1.2 million died of chronic HBV infection each year [2]. As is known to all, the HBV infection is usually complex and variable [3], and can result in different clinical outcomes. Several progressive stages are confirmed for chronic HBV infection,including chronic hepatitis B (CHB), liver cirrhosis (LC), as well as hepatocellular carcinoma (HCC) [4]. Chronic HBV infection can progress into CHB, while about 10%-30% will progress to liver cirrhosis and HCC [5]. Thus, the degree of chronic HBV infection varies enormously among individuals, which represents a complex biological process where the cellular mechanisms and genetic contributions of pathogenesis remain unknown [6, 7]. These facts contribute to the development of more personalized therapy, diagnosis or prognosis, which then reduce the health disparity among the victims.

Viral factors (genotype and mutations) [8], host factors and environmental factors [9] are considered to involve in the disease progression of HBV infection, from HBV clearance to chronic infection that may progress into liver cirrhosis and HCC [10-12]. To date, several host factors are available, including age of infection, gender, volume of alcohol intake, obesity, smoking, diabetes, chemical exposure and chemical exposure [13, 14]. In addition, results from twin studies and candidate gene approaches demonstrated that host genetic factors may be closely associated with the outcome of HBV infection and progression [15-17]. Single nucleotide polymorphisms (SNPs), representing the most common type of genetic variation in human beings, may change the structure and biological function of the encoded protein [18]. Recently, genetic polymorphisms have attracted more attention due to their etiological roles in defining the disease progression of HBV infection. Recent studies indicated that variants in some host genes, including interleukin-4 (IL-4) gene *-2590C/T* (*rs2243250*) and *-233C/T* (*rs20708742590*) [19], tumor necrosis factor-α (TNF-α) gene *-308 G/A* [20], toll-like receptor 3 (TLR3) gene (*rs1879026* and *rs3775290*) [21], vascular endothelial growth factor (VEGF) gene *634 G/C* (*rs2010963*) [22] and cytotoxic T-lymphocyte-associated antigen 4 (CTLA-4) gene *+49A/G* [23], were associated with persistent HBV infection and natural clearance.

In the past few years, several genome-wide association studies (GWAS) have identified that SNPs proximate to the HLA-DP, HLA-DQ, and HLA-DR loci are significantly correlated with HBV infection outcomes [24-26]. Additionally, several studies on different populations have focused on the roles of *HLA-DQ* gene polymorphism in the pathogenesis of HBV infection. However, these findings are still controversial. Furthermore, a single-center study may have an inadequate sample size and lack of statistical power to obtain reliable conclusions. In this study, a comprehensive meta-analysis was utilized to precisely evaluate the correlation between *HLA-DQ* gene polymorphism and HBV infection complications (e.g. CHB, LC, and HCC).

## RESULTS

### Study characteristics

**Figure 1:**
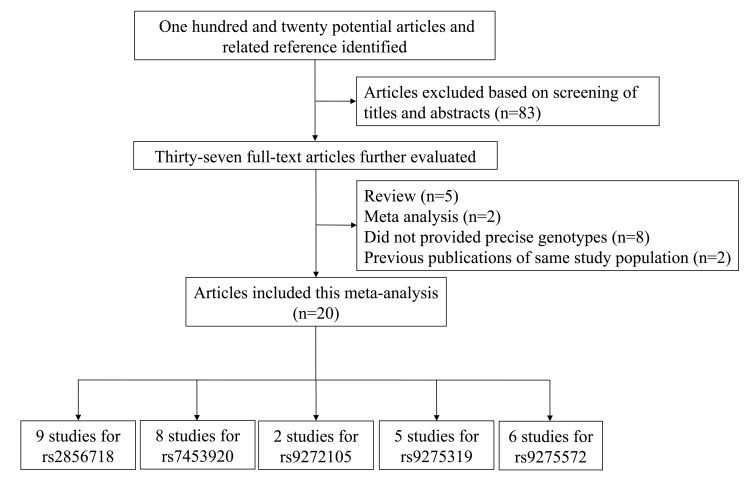
The flow charts of literature search and study selection

**Figure 2:**
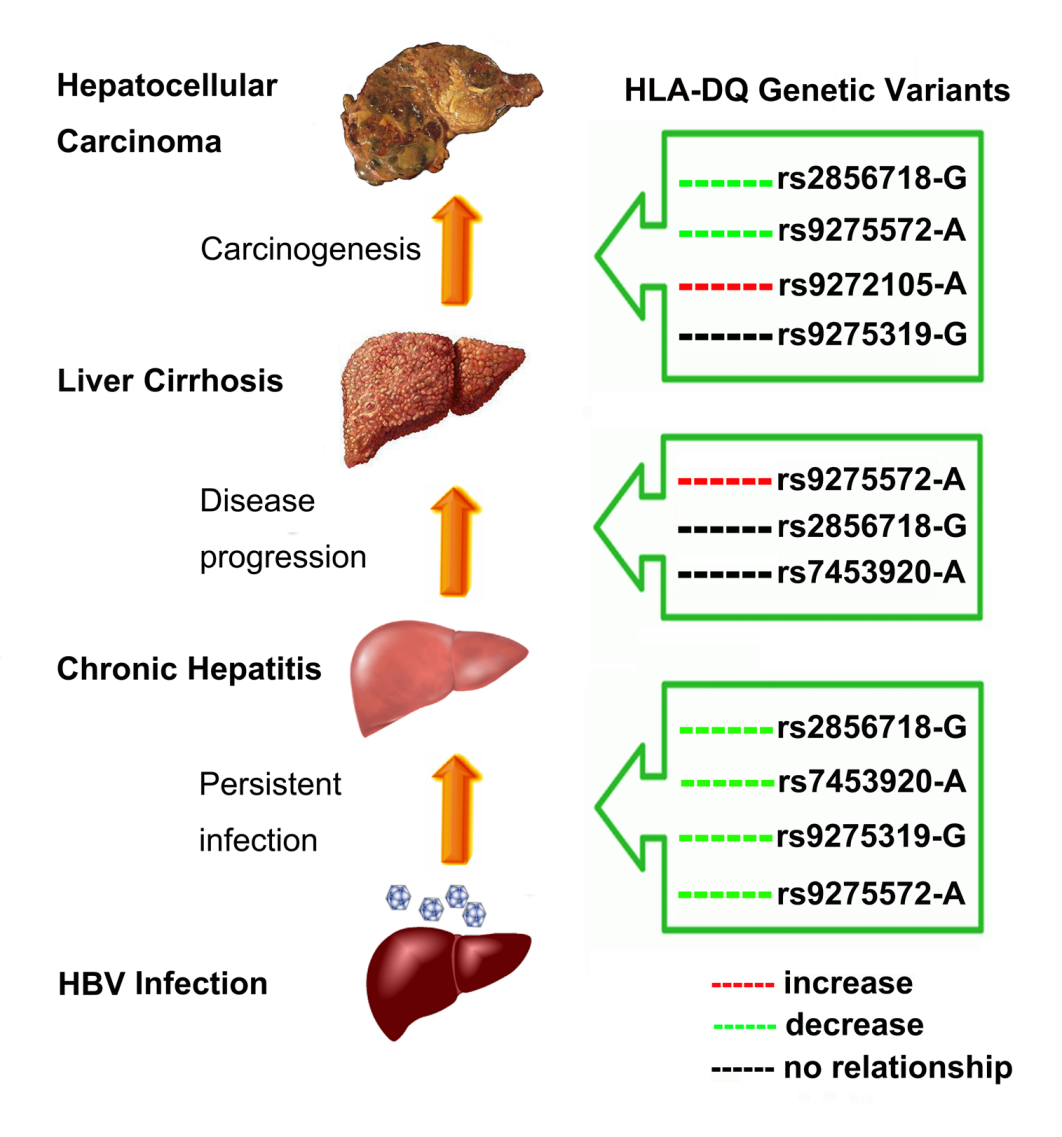
Host HLA-DQ region polymorphisms influencing infection outcomes

According to our search strategy, 120 publications were identified through the initial search after excluding 83 articles. A flow diagram of the detail selection and exclusion process was displayed in Figure 1. After full review, 37 studies were then excluded based on the following aspects: duplicate data, review articles, meta-analyses and case-only studies. Finally, 20 studies (28347 cases and 37329 controls) were chosen, and the data were extracted. Among these publications, there were 9 studies for rs2856718 [24, 25, 28, 31-33, 40, 43, 44], 8 for rs7453920 [24, 25, 28, 31, 34. 39, 44, 45], 2 for rs9272105 [29, 38], 5 for rs9275319 [33, 36-38, 40] and 6 for rs9275572 [30-32, 35, 41, 43]. The main features of each eligible study were summarized in Table 1 and Figure 2, respectively.

**Table1:** Characteristics of the studies included in the meta-analysis

Study	Year	Ethnicity	Subgroup	Genotyping method	Case	Control	No. of cases	No. of controls	Polymorphisms	NOS
Mbarek H [24]	2011	Japanese	GWAS	GeneChip	CHB	non-HBV	458	2056	rs2856718; rs7453920	6
	2011	Japanese	First replication	Invader assay	CHB	non-HBV	606	2023		
	2011	Japanese	Second replication	TaqMan	CHB	non-HBV	379	1539		
	2011	Japanese	Third replication	TaqMan	CHB	non-HBV	1226	879		
Hu LM [28]	2012	Chinese		TaqMan	HBV Carriers; HBV-HCC	HBV clearance	2644	1344	rs2856718; rs7453920	7
Li SP [29]	2012	Chinese	GWAS Southern	Gene Chip	HCC	HBV positive	1075	990	rs9272105	7
	2012	Chinese	GWAS Central	Gene Chip	HCC	HBV positive	500	500		
	2012	Chinese	Validation 1	iPLEX/TaqMan	HCC	HBV-positive	2112	2208		
	2012	Chinese	Validation 2	iPLEX/TaqMan	HCC	HBV-positive	1021	1491		
	2012	Chinese	Replication	iPLEX/TaqMan	HCC	HBV-positive	1298	1026		
Hu ZB [25]	2013	Chinese	GWAS	GeneChip	HBV carriers	HBV clearance	951	937	rs7453920 rs2856718	6
	2013	Chinese	Replication Ia	iPLEX	HBV carriers	HBV clearance	1248	1248	rs7453920 rs2856718	
	2013	Chinese	Replication Ib	TaqMan	HBV carriers	HBV clearance	1000	1803	rs7453920 rs2856718	
	2013	Chinese	Replication IIa	iPLEX	HBV carriers	HBV clearance	981	1417	rs7453920	
	2013	Chinese	Replication IIb	TaqMan	HBV carriers	HBV clearance	1001	1205	rs7453920	
Chen KM [30]	2013	Chinese		TaqMan	HCC	CHB	506	772	rs9275572	8
Al-Qahtani AA [31]	2014	Saudi Arabian		PCR-based genotyping/ TaqMan	HBV carriers (AsC, LC, HCC)	Healthy controls, HBV clearance	781	302, 587	rs2856718; rs7453920; rs9275572	7
Zhang X [32]	2014	Chinese		Flight mass spectrometry	HBV carriers (CHB, LC, HCC);	Healthy controls, HBV clearance	792	507, 350	rs2856718; rs9275572	8
Ji XW [33]	2014	Chinese		Real-time PCR	HBV carriers (CHB, ASCs, LC)	Healthy controls; HBV clearance	2489	1342; 327	rs2856718; rs9275319	8
Liao Y [34]	2014	Chinese		HRM	chronic HBV carriers; HCC	Healthy controls; HBV clearance	677	237, 398	rs7453920	8
Hou SH [35]	2015	Chinese		TaqMan	HBV carriers (CHB, LC, HCC)	Healthy controls; HBV clearance	310	316, 295	rs9275572	8
Hou SH [36]	2015	Chinese		TaqMan	HBV carriers (CHB, LC, HCC)	Healthy controls; HBV clearance	310	316, 295	rs9275319	8
Kim LH [37]	2015	Korean		TaqMan	CHB; HCC	Population control samples	958	2880	rs9275319	7
Wen J [38]	2015	Chinese		TaqMan	HCC	HBV persistent carriers	1507	1560	rs9272105; rs9275319	6
Liao Y [39]	2015	Chinese	Tibetans	HRM	HBV carriers	HBV clearance	422	486	rs7453920	7
		Chinese	Uygurs	HRM	HBV carriers	HBV clearance	195	235		
Jiang DK [40]	2015	Chinese	Shanghai	MassARRAY/TaqMan	LC	CHB	440	1265	rs9275319	6
		Chinese	Beijing		LC	CHB	272	1336		
Liu WX [41]	2016	Chinese		Flight mass spectrometry	HBV carriers	Healthy controls; HBV clearance	396	254, 175	rs2856718; rs9275572	8
Fan JH [42]	2016	Chinese		MassARRAY	HBV carriers	Healthy controls; HBV clearance	397	238, 434	rs9275319	8
Gao X [43]	2016	Chinese		Flight mass spectrometry	HBV carriers (CHB, LC, HCC)	Healthy controls	784	507	rs2856718; rs9275572	8
Trinks [44]	2017	Argentinean	Central areas	TaqMan	HBV carriers	Healthy controls; HBV clearance	201	207, 318	rs2856718; rs7453920	8
	2017	Argentinean	North-western areas	TaqMan	HBV carriers		200	201, 313		
Pereira VRZB [45]	2017	Brazilian		TaqMan	CHB	Healthy controls	210	210	rs7453920	8

CHB: Chronic Hepatitis B; HBV: Hepatitis B Virus; HCC: Hepatocellular Carcinoma; LC: Liver Cirrhosis; AsC: Asymptomatic Carriers; NOS: Newcastle-Ottawa Scale.

### Association between *HLA-DQ rs2856718* polymorphism and outcome of HBV infection

**Figure 3:**
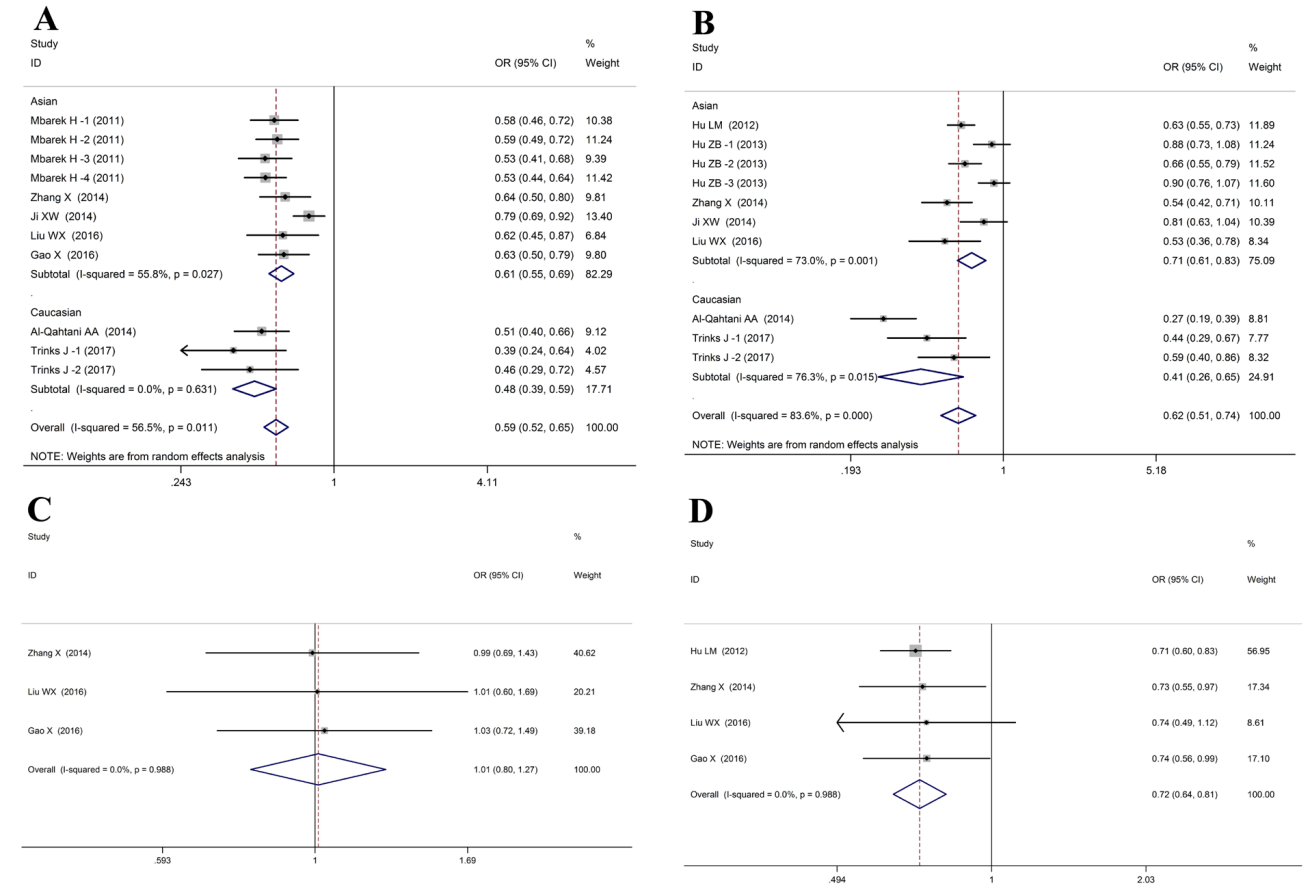
Forest plots for HLA-DQ rs2856718 polymorphism and HBV infection outcomes **A**. HBV infection vs. Control (AA vs. AG+GG); **B**. HBV infection vs. NC (AA vs. AG+GG); **C**. LC vs. CHB (AA vs. AG+GG); **D**. HCC vs. LC+CHB (AA vs. AG+GG).

In the meta-analysis, 9 studies including 14155 cases and 17219 controls were included to investigate the associations between *HLA-DQ rs2856718* polymorphism and HBV infection outcomes (Table 2). These results indicated that *HLA-DQ rs2856718* was considered to be associated with a decrease of HBV infection risk (HBV infection vs. Control: allele: OR= 0.66, 95%CI: 0.60-0.73, *P*_Z_ < 0.01; heterozygous: OR= 0.66, 95%CI: 0.62-0.71, *P*_Z_ < 0.01; homozygous: OR= 0.46, 95%CI: 0.37-0.55, *P*_Z_ < 0.01; recessive: OR= 0.60, 95%CI: 0.50-0.72, *P*_Z_ < 0.01; dominant: OR= 0.59, 95%CI: 0.52-0.65, *P*_Z_ < 0.01, Figure 3A). Whereas, in the Caucasian populations, no association was noticed in the recessive model (GG vs. AG+AA: OR = 0.63, 95%CI: 0.38-1.06, *P*_Z_ = 0.08). Meanwhile, *HLA-DQ rs2856718* polymorphism showed significant association with HBV clearance (HBV infection vs. SC: allele: OR= 0.74, 95%CI: 0.67-0.82, *P*_Z_ < 0.01; heterozygous: OR= 0.63, 95%CI: 0.51-0.79, *P*_Z_ < 0.01; homozygous: OR= 0.74, 95%CI: 0.70-0.78, *P*_Z_ < 0.01; recessive: OR= 0.74, 95%CI: 0.63-0.87, *P*_Z_ < 0.01; dominant: OR= 0.62, 95%CI: 0.51-0.74, *P*_Z_ < 0.01, Figure 3B). Moreover, the HLA-DQ rs2856718 polymorphism was correlated with a decrease of HBV-related HCC risk in four genetic models (HCC vs. LC+CHB: allele: OR = 0.80, 95%CI: 0.76-0.90, *P*_Z_ < 0.01; heterozygous: OR = 0.71, 95%CI: 0.63-0.81, *P*_Z_ < 0.01; homozygous: OR = 0.74, 95%CI: 0.62-0.88, *P*_Z_ < 0.01; dominant: OR = 0.72, 95%CI: 0.64-0.81, *P*_Z_ < 0.01, Figure 3D). However, no association was noticed between *HLA-DQ rs2856718* polymorphism and LC development from CHB in all genetic models (LC vs. CHB: allele: OR= 0.99, 95%CI: 0.84-1.17, *P*_Z_ = 0.88; heterozygous: OR= 1.03, 95%CI: 0.81-1.32, *P*_Z_ = 0.81; homozygous: OR = 0.96, 95%CI: 0.67-1.34, *P*_Z_ = 0.78; recessive: OR= 0.94, 95%CI: 0.68-1.28, *P*_Z_ = 0.69; dominant: OR= 1.01, 95%CI: 0.80-1.27, *P*_Z_ = 0.93, Figure 3C).

**Table 2:** Main results of the meta-analysis of the association between HLA-DQ rs2856718 polymorphism and HBV infection outcomes

Comparison	Subgroup	Allele model (G vs. A）			Heterozygous model (AG vs. AA）			Homozygous model (GG vs. AA）			Recessive model (GG vs. AG+AA）			Dominant model (AG+GG vs. AA）		
		OR (95%CI)	*P*_H_	*P*_Z_	OR (95%CI)	*P*_H_	*P*_Z_	OR (95%CI)	*P*_H_	*P*_Z_	OR (95%CI)	*P*_H_	*P*_Z_	OR (95%CI)	*P*_H_	*P*_Z_
HBV infection vs. Control	Overall	0.66 (0.60-0.73)	<0.01	<0.01	0.66 (0.62-0.71)	0.04	<0.01	0.46 (0.37-0.55)	<0.01	<0.01	0.60 (0.50-0.72)	<0.01	<0.01	0.59 (0.52-0.65)	0.01	<0.01
	Asian	0.67 (0.60-0.75)	<0.01	<0.01	0.70 (0.64-0.75)	0.49	<0.01	0.46 (0.37-0.58)	<0.01	<0.01	0.58 (0.48-0.71)	<0.01	<0.01	0.61 (0.55-0.69)	0.03	<0.01
	Caucasian	0.63 (0.50-0.78)	0.08	<0.01	0.46 (0.37-0.58)	0.70	<0.01	0.41 (0.24-0.70)	0.02	<0.01	0.63 (0.38-1.06)	<0.01	0.08	0.48 (0.39-0.59)	0.63	<0.01
HBV infection vs. NC	Overall	0.74 (0.67-0.82)	<0.01	<0.01	0.63 (0.51-0.79)	<0.01	<0.01	0.74 (0.70-0.78)	<0.01	<0.01	0.74 (0.63-0.87)	0.04	<0.01	0.62 (0.51-0.74)	<0.01	<0.01
	Asian	0.78 (0.70-0.87)	<0.01	<0.01	0.75 (0.66-0.87)	0.01	<0.01	0.75 (0.71-0.80)	<0.01	<0.01	0.74 (0.64-0.84)	<0.01	<0.01	0.71 (0.61-0.83)	<0.01	<0.01
	Caucasian	0.64 (0.56-0.73)	0.64	<0.01	0.41 (0.18-0.90)	<0.01	0.03	0.65 (0.57-0.70)	0.56	<0.01	0.72 (0.38-1.38)	<0.01	0.33	0.41 (0.26-0.65)	0.02	<0.01
LC vs. CHB	Chinese	0.99 (0.84-1.17)	0.99	0.88	1.03 (0.81-1.32)	0.99	0.81	0.96 (0.67-1.34)	0.99	0.78	0.94 (0.68-1.28)	0.99	0.69	1.01 (0.80-1.27)	0.99	0.93
HCC vs. LC+CHB	Chinese	0.80 (0.76-0.90)	0.90	<0.01	0.71 (0.63-0.81)	0.85	<0.01	0.74 (0.62-0.88)	0.83	<0.01	0.90 (0.77-1.06)	0.52	0.20	0.72 (0.64-0.81)	0.99	<0.01

-*Because there was only one study with this genotype of rs2856718, the value could not be calculated.

### Meta-analysis for *HLA-DQ rs9275572* polymorphism with HBV infection outcomes

**Figure 4:**
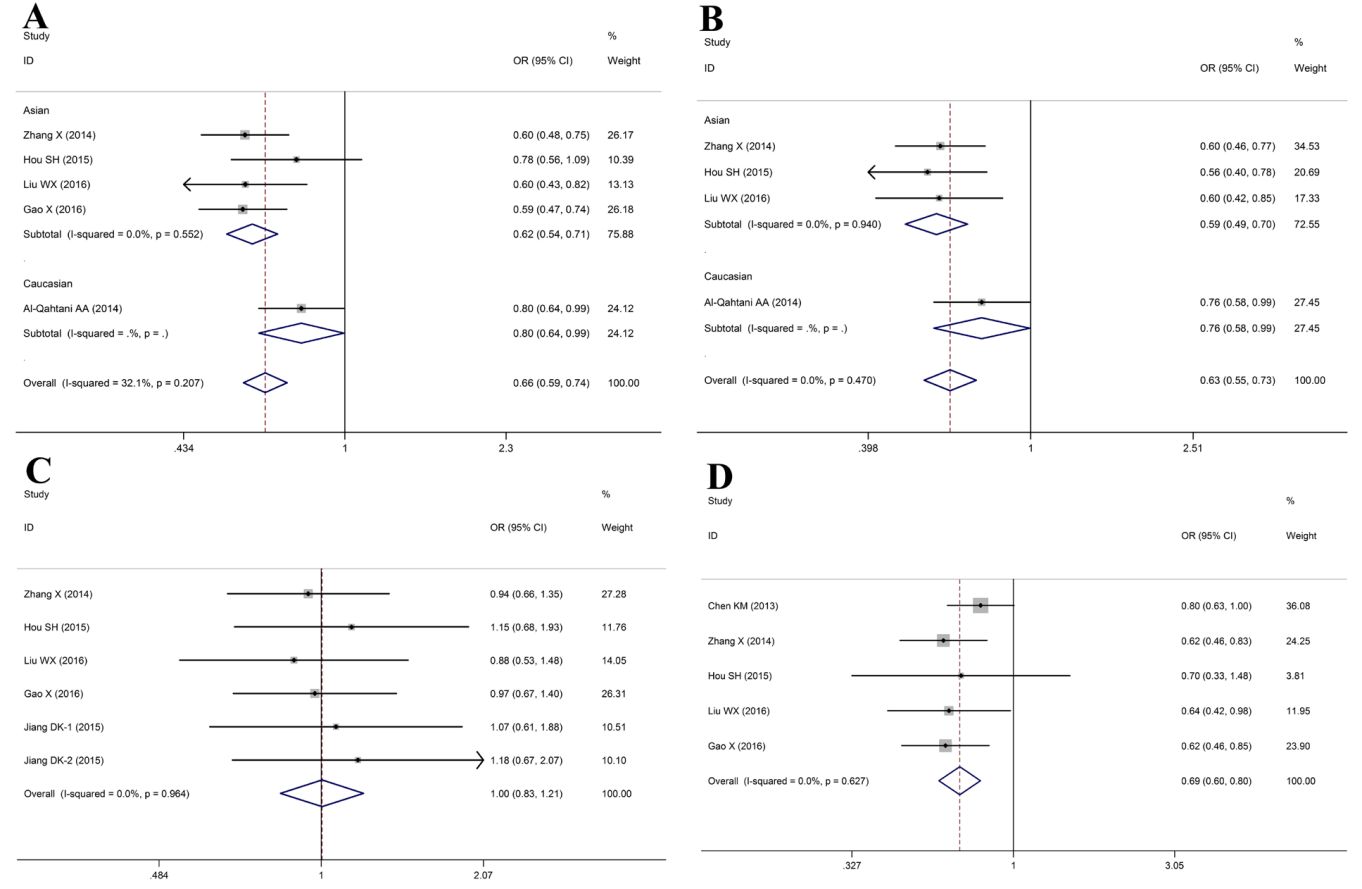
Forest plots for HLA-DQ rs9275572 polymorphism and HBV infection outcomes **A**. HBV infection vs. Control (AA vs. AG+GG); **B**. HBV infection vs. NC (AA vs. AG+GG); **C**. LC vs. CHB (AA vs. AG+GG); **D**. HCC vs. LC+CHB (AA vs. AG+GG)

Finally, 6 studies including 3569 cases and 4065 controls were subject to analysis using fixed-effects or random-effects model (Table 3). Pooled analysis demonstrated that *HLA-DQ rs9275572* polymorphism was correlated with a significantly increased risk of HBV infection in total population (HBV infection vs. Control: A vs. G: OR = 0.68, 95%CI: 0.62-0.74, *P*_Z_ < 0.01; AG vs. GG: OR = 0.73, 95%CI: 0.65-0.82, *P*_Z_ < 0.01; AA vs. GG: OR = 0.45, 95%CI: 0.37-0.56, *P*_Z_ < 0.01; AA vs. AG+GG: OR = 0.51, 95%CI: 0.42-0.62, *P*_Z_ < 0.01; AA+AG vs. GG: OR = 0.66, 95%CI: 0.59-0.74, *P*_Z_ < 0.01, Figure 4A). With regards to the HBV clearance, our data indicated that subjects with the *HLA-DQ rs9275572-A* allele showed a significantly lower incidence of spontaneous clearance after HBV infection (HBV infection vs. NC: allele: OR = 0.70, 95%CI: 0.62-0.79, *P*_Z_ < 0.01; heterozygous: OR = 0.65, 95%CI: 0.56-0.76, *P*_Z_ < 0.01; homozygous: OR = 0.57, 95%CI: 0.44-0.76, *P*_Z_ < 0.01; recessive: OR = 0.67, 95%CI: 0.52-0.87, *P*_Z_ < 0.01; dominant: OR = 0.63, 95%CI: 0.55-0.73, *P*_Z_ < 0.01, Figure 4B). Among LC and CHB patients, we found a significant relationship between the A allele and decreased risk of CHB to LC with an OR of 1.34 (95%CI: 1.14-1.56) for HLA-DQ rs9275572, but there was no significant correlation in the heterozygous model (OR = 0.97, 95%CI: 0.80-1.18, *P*_Z_ = 0.76), homozygous model (OR = 1.22, 95%CI: 0.83-1.80, *P*_Z_ = 0.31), recessive model (OR = 1.25, 95%CI: 0.85-1.82, *P*_Z_ = 0.25) and dominant model (OR = 1.00, 95%CI: 0.83-1.21, *P*_Z_ = 0.97, Figure 4C). These results revealed that a significant correlation might be presented between the *HLA-DQ rs9275572* polymorphism and HBV-related HCC in all gene model (HCC vs. LC+CHB: allele: OR = 0.71, 95%CI: 0.63-0.80, *P*_Z_ < 0.01; heterozygous: OR = 0.73, 95%CI: 0.64-0.84, *P*_Z_ < 0.01; homozygous: OR = 0.49, 95%CI: 0.35-0.68, *P*_Z_ < 0.01; recessive: OR = 0.54, 95%CI: 0.39-0.76, *P*_Z_ < 0.01; dominant: OR = 0.69, 95%CI: 0.60-0.80, *P*_Z_ < 0.01, Figure 4D).

**Table 3:** Main results of the meta-analysis of the association between HLA-DQ rs9275572 polymorphism and HBV infection outcomes

Comparison	Subgroup	Allele model (A vs. G)			Heterozygous model (AG vs. GG)			Homozygous model (AA vs. GG)			Recessive model (AA vs. AG+GG)			Dominant model (AA+AG vs. GG)		
		OR (95%CI)	*P*_H_	*P*_Z_	OR (95%CI)	*P*_H_	*P*_Z_	OR (95%CI)	*P*_H_	*P*_Z_	OR (95%CI)	*P*_H_	*P*_Z_	OR (95%CI)	*P*_H_	*P*_Z_
HBV infection vs. Control	Overall	0.68 (0.62-0.74)	0.11	<0.01	0.73 (0.65-0.82)	0.34	<0.01	0.45 (0.37-0.56)	0.23	<0.01	0.51 (0.42-0.62)	0.41	<0.01	0.66 (0.59-0.74)	0.21	<0.01
	Asian	0.64 (0.54-0.71)	0.34	<0.01	0.68 (0.59-0.78)	0.89	<0.01	0.40 (0.31-0.52)	0.35	<0.01	0.47 (0.37-0.60)	0.46	<0.01	0.62 (0.54-0.71)	0.55	<0.01
	Caucasian	0.78 (0.66-0.91)	-*	<0.01	0.89 (0.71-1.13)	-*	0.33	0.56 (0.40-0.78)	-*	<0.01	0.59 (0.43-0.81)	-*	<0.01	0.80 (0.64-0.99)	-*	0.04
HBV infection vs. NC	Overall	0.70 (0.62-0.79)	0.61	<0.01	0.65 (0.56-0.76)	0.30	<0.01	0.57 (0.44-0.76)	0.99	<0.01	0.67 (0.52-0.87)	0.99	<0.01	0.63 (0.55-0.73)	0.47	<0.01
	Asian	0.66 (0.57-0.76)	0.91	<0.01	0.59 (0.49-0.71)	0.92	<0.01	0.57 (0.39-0.82)	0.99	<0.01	0.70 (0.49-1.00)	0.99	0.05	0.59 (0.49-0.70)	0.94	<0.01
	Caucasian	0.78 (0.64-0.94)	-*	0.01	0.82 (0.61-1.09)	-*	0.17	0.58 (0.38-0.88)	-*	0.01	0.65 (0.44-0.95)	-*	0.03	0.76 (0.58-0.99)	-*	0.05
LC vs. CHB	Chinese	1.34 (1.14-1.56)	0.99	<0.01	0.97 (0.80-1.18)	0.82	0.76	1.22 (0.83-1.80)	0.82	0.31	1.25 (0.85-1.82)	0.73	0.25	1.00 (0.83-1.21)	0.96	0.97
HCC vs. LC+CHB	Chinese	0.71 (0.63-0.80)	0.79	<0.01	073 (0.64-0.84)	0.55	<0.01	0.49 (0.35-0.68)	1.00	<0.01	0.54 (0.39-0.76)	0.99	<0.01	0.69 (0.60-0.80)	0.63	<0.01

-*Because there was only one study with this genotype of rs9275572, the value could not be calculated.

### Association between *HLA-DQ rs7453920* polymorphism and HBV infection outcome

**Figure 5:**
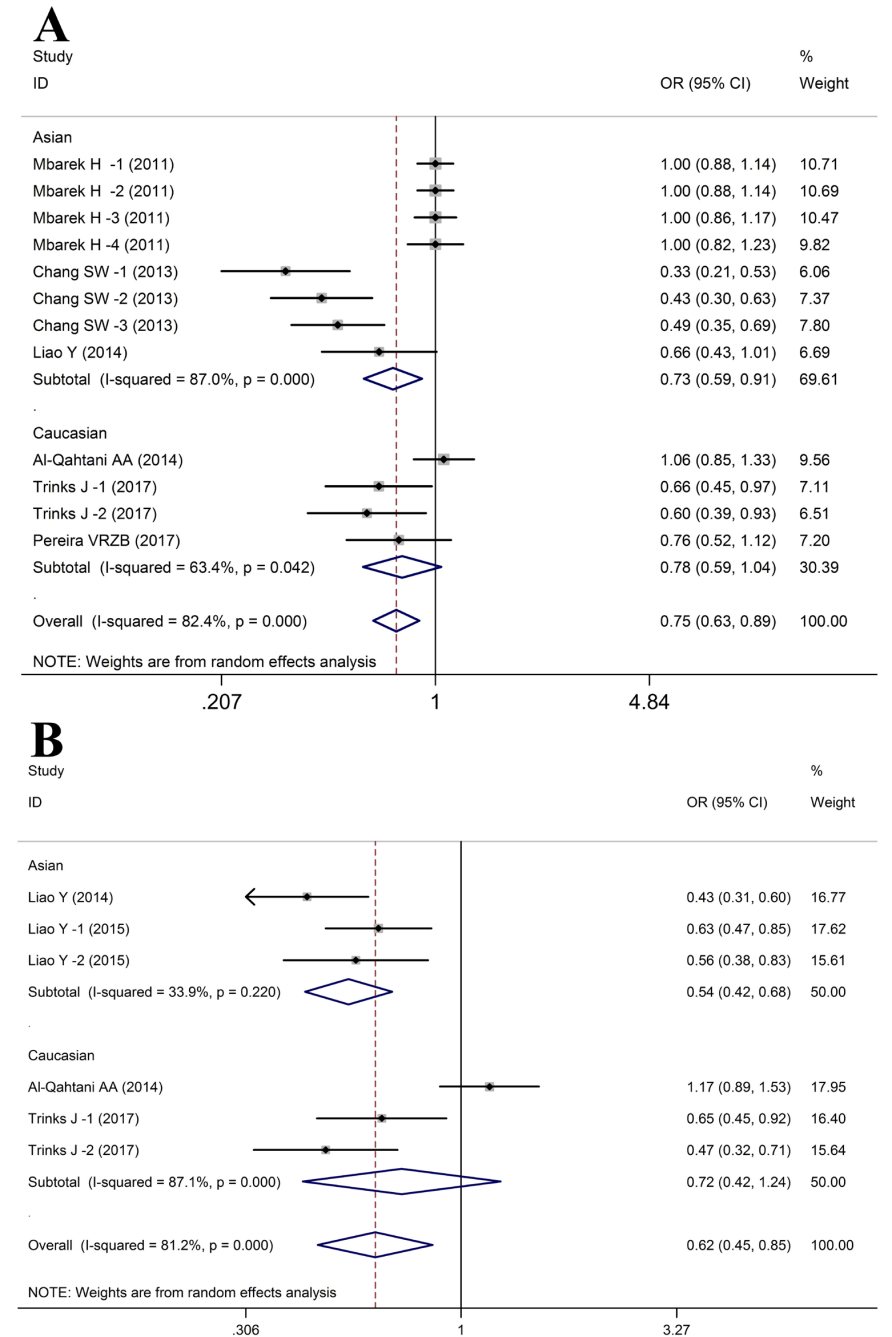
Forest plots for HLA-DQ rs7453920 polymorphism and HBV infection outcomes **A**. HBV infection vs. Control (AA vs. AG+GG); **B**. HBV infection vs. NC (AA vs. AG+GG).

In this meta-analysis, *HLA-DQ rs7453920* polymorphism was confirmed to be significantly associated with HBV infection in the following genetic models (HBV infection vs. Control: A vs. G: OR = 0.72, 95%CI: 0.62-0.82, *P*_Z_ < 0.01; AG vs. GG: OR = 0.76, 95%CI: 0.64-0.89, *P*_Z_ < 0.01; AA+AG vs. GG: OR = 0.75, 95%CI: 0.63-0.89, *P*_Z_ < 0.01, Figure 5A). In contrast, no significant correlation was identified between *HLA-DQ rs7453920* polymorphism and HBV infection outcome in the Homozygous model (OR = 0.96, 95%CI: 0.82-1.12, *P*_Z_ = 0.58) and Recessive model (OR = 0.99, 95%CI: 0.85-1.15, *P*_Z_ = 0.90) (Table 4). Meanwhile, we confirmed that *HLA-DQ rs7453920* polymorphism was associated with HBV clearance in total population (HBV infection vs. NC: allele: OR = 0.64, 95%CI: 0.40-1.02, *P*_Z_ < 0.01; heterozygous: OR = 0.62, 95%CI: 0.45-0.85, *P*_Z_ < 0.01; dominant: OR = 0.62, 95%CI: 0.45-0.85, *P*_Z_ < 0.01, Figure 5B).

**Table 4:** Main results of the meta-analysis of the association between HLA-DQ rs7453920 polymorphism and HBV infection outcomes

Comparison	Subgroup	Allele model (A vs. G)			Heterozygous model (AG vs. GG)			Homozygous model (AA vs. GG)			Recessive model (AA vs. AG+GG)			Dominant model (AA+AG vs. GG)		
		OR (95%CI)	*P*_H_	*P*_Z_	OR (95%CI)	*P*_H_	*P*_Z_	OR (95%CI)	*P*_H_	*P*_Z_	OR (95%CI)	*P*_H_	*P*_Z_	OR (95%CI)	*P*_H_	*P*_Z_
HBV infection vs. Control	Overall	0.72 (0.62-0.82)	<0.01	<0.01	0.76 (0.64-0.89)	<0.01	<0.01	0.96 (0.82-1.12)	0.12	0.58	0.99 (0.85-1.15)	0.22	0.90	0.75 (0.63-0.89)	<0.01	<0.01
	Asian	0.71 (0.59-0.85)	<0.01	<0.01	0.75 (0.61-0.92)	<0.01	<0.01	0.91 (0.75-1.11)	0.13	0.34	0.91 (0.75-1.11)	0.19	0.38	0.73 (0.59-0.91)	<0.01	<0.01
	Caucasian	0.72 (0.60-0.87)	<0.01	<0.01	0.77 (0.60-1.00)	0.13	0.05	1.04 (0.81-1.35)	0.19	0.75	1.11 (0.88-1.44)	0.38	0.39	0.78 (0.59-1.04)	0.04	0.09
HBV infection vs. NC	Overall	0.64 (0.40-1.02)	<0.01	0.06	0.62 (0.47-0.81)	<0.01	<0.01	0.69 (0.35-1.37)	<0.01	0.29	0.79 (0.43-1.47)	<0.01	0.46	0.62 (0.45-0.85)	<0.01	<0.01
	Asian	0.55 (0.46-0.65)	0.42	<0.01	0.54 (0.40-0.73)	0.11	<0.01	0.51 (0.20-1.30)	0.16	0.16	0.59 (0.24-1.46)	0.17	0.26	0.54 (0.42-0.68)	0.22	<0.01
	Caucasian	0.76 (0.33-1.73)	<0.01	0.51	0.70 (0.45-1.08)	0.01	0.10	0.83 (0.33-2.08)	<0.01	0.70	0.96 (0.44-2.07)	0.01	0.92	0.72 (0.42-1.24)	<0.01	0.24

### Meta-analysis for *HLA-DQ rs9275319* polymorphism with HBV infection outcomes

**Figure 6:**
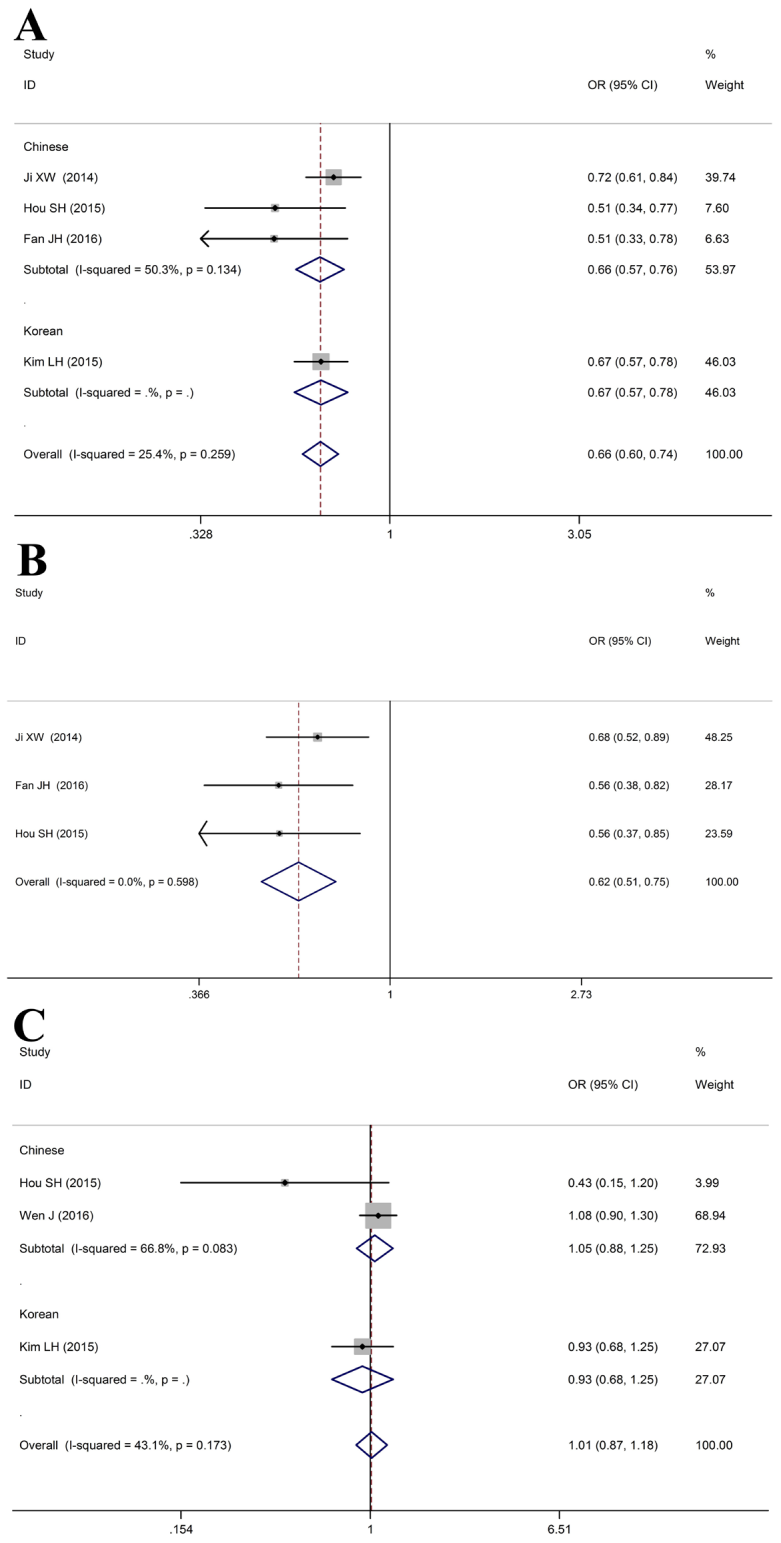
Forest plots for HLA-DQ rs9275319 polymorphism and HBV infection outcomes **A**. HBV infection vs. Control (AA vs. AG+GG); **B**. HBV infection vs. NC (AA vs. AG+GG); C. HCC vs. LC+CHB (AA vs. AG+GG).

As shown in Table 5, Logistic regression analysis revealed a significant correlation between *HLA-DQ rs9275319* polymorphism and a reduced risk of HBV infection in the HBV infection group (HBV infection vs. Control: allele: OR = 0.68, 95%CI: 0.62-0.74, *P*_Z_ < 0.01; heterozygous: OR = 0.69, 95%CI: 0.62-0.77, *P*_Z_ < 0.01; homozygous: OR = 0.50, 95%CI: 0.38-0.65, *P*_Z_ < 0.01; recessive: OR = 0.55, 95%CI: 0.42-0.72, *P*_Z_ < 0.01; dominant: OR = 0.66, 95%CI: 0.60-0.74, *P*_Z_ < 0.01), as compared to healthy controls (Figure 6A). Meanwhile, *HLA-DQ rs9275319* polymorphism was significantly associated with HBV clearance (HBV infection vs. NC: G vs. A: OR = 0.6, 95%CI: 0.54-0.76, *P*_Z_ < 0.01; AG vs. AA: OR = 0.63, 95%CI: 0.51-0.77, *P*_Z_ < 0.01; GG vs. AA: OR = 0.52, 95%CI: 0.28-0.95, *P*_Z_ = 0.03; AG+GG vs. AA: OR = 0.62, 95%CI: 0.51-0.75, *P*_Z_ < 0.01) (Figure 6B). However, no association was observed between *HLA-DQ rs9275319* polymorphism and HBV-related HCC (HCC vs. LC+CHB: allele: OR = 0.99, 95%CI: 0.86-1.14, *P*_Z_ = 0.92; heterozygous: OR = 1.04, 95%CI: 0.44-1.21, *P*_Z_ = 0.67; homozygous: OR = 0.81, 95%CI: 0.50-1.32, *P*_Z_ = 0.40; recessive: OR = 0.82, 95%CI: 0.51-1.32, *P*_Z_ = 0.88; dominant: OR = 1.01, 95%CI: 0.87-1.18, *P*_Z_ = 0.87, Figure 6C).

**Table 5:** Main results of the meta-analysis of the association between HLA-DQ rs9275319 polymorphism and HBV infection outcomes

Comparison	Subgroup	Allele model (G vs. A）			Heterozygous model (AG vs. AA）			Homozygous model (GG vs. AA）			Recessive model (GG vs. AG+AA）			Dominant model (AG+GG vs. AA）		
		OR (95%CI)	*P*_H_	*P*_Z_	OR (95%CI)	*P*_H_	*P*_Z_	OR (95%CI)	*P*_H_	*P*_Z_	OR (95%CI)	*P*_H_	*P*_Z_	OR (95%CI)	*P*_H_	*P*_Z_
HBV infection vs. Control	Overall	0.68 (0.62-0.74)	0.12	<0.01	0.69 (0.62-0.77)	0.35	<0.01	0.50 (0.38-0.65)	0.16	<0.01	0.55 (0.42-0.72)	0.18	<0.01	0.66 (0.60-0.74)	0.26	<0.01
	Chinese	0.69 (0.61-0.79)	0.06	<0.01	0.66 (0.57-0.77)	0.27	<0.01	0.66 (0.42-1.03)	0.20	0.07	0.71 (0.46-1.12)	0.23	0.140	0.66 (0.57-0.76)	0.13	<0.01
	Korean	0.66 (0.8-0.76)	-*	<0.01	0.73 (0.62-0.86)	-*	<0.01	0.44 (0.31-0.62)	-*	<0.01	0.49 (0.35-0.68)	-*	<0.01	0.67 (0.57-0.78)	-*	<0.01
HBV infection vs. NC	Chinese	0.64 (0.54-0.76)	0.39	<0.01	0.63 (0.51-0.77)	0.78	<0.01	0.52 (0.28-0.95)	0.45	0.03	0.57 (0.37-1.04)	0.45	0.07	0.62 (0.51-0.75)	0.60	<0.01
HCC vs. LC+CHB	Overall	0.99 (0.86-1.14)	0.19	0.92	1.04 (0.44-1.21)	0.18	0.67	0.81 (0.50-1.32)	0.90	0.40	0.82 (0.51-1.32)	0.88	0.41	1.01 (0.87-1.18)	0.17	0.87
	Chinese	1.02 (0.86-1.20)	0.09	0.84	1.07 (0.89-1.29)	0.09	0.45	0.73 (0.37-1.43)	0.90	0.36	0.72 (0.37-1.42)	0.95	0.35	1.05 (0.88-1.25)	0.08	0.62
	Korean	0.94 (0.72-1.21)	-*	0.61	0.93 (0.67-1.28)	-*	0.65	0.91 (0.46-1.82)	-*	0.80	0.93 (0.47-1.85)	-*	0.84	0.93 (0.68-1.25)	-*	0.62

-*Because there was only one study with this genotype of rs9275319, the value could not be calculated.

### Meta-analysis for *HLA-DQ rs9272105* polymorphism with HBV infection outcomes

**Figure 7:**
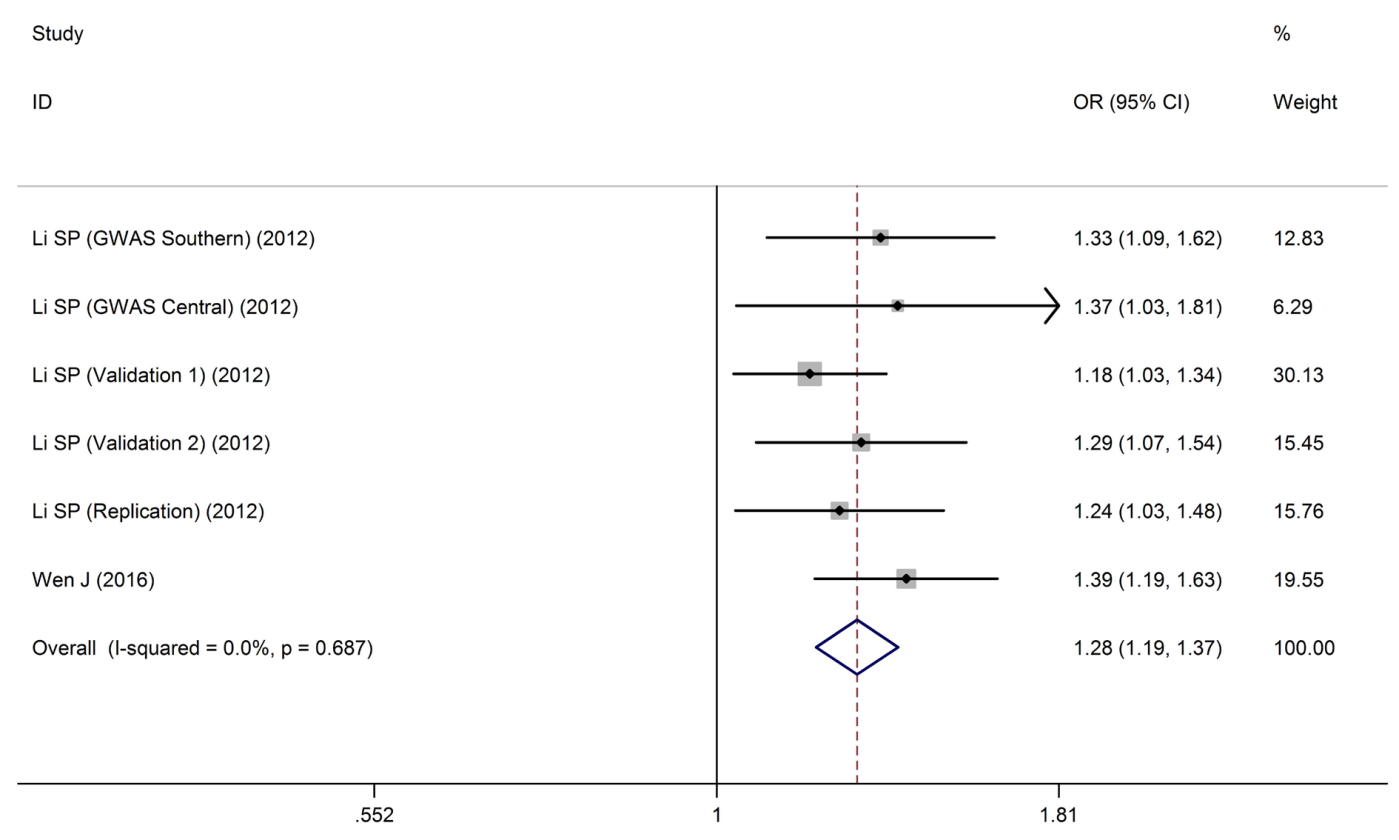
Forest plots for HLA-DQ rs9272105 polymorphism and HBV infection outcomes HCC vs. LC+CHB (AA vs. AG+GG).

As shown in Table 6, *HLA-DQ rs9272105* polymorphism was significantly associated with HBV-related HCC in all gene models (HCC vs. (LC+CHB): allele: OR = 1.31, 95%CI: 1.25-1.37, *P*_Z_ < 0.01; heterozygous: OR = 1.11, 95%CI: 1.03-1.20, *P*_Z_ < 0.01; homozygous: OR = 1.70, 95%CI: 1.56-1.86, *P*_Z_ < 0.01; recessive: OR = 1.59, 95%CI: 1.48-1.72, *P*_Z_ < 0.01; dominant: OR = 1.28, 95%CI: 1.19-1.37, *P*_Z_ < 0.01, Figure 7).

**Table 6:** Main results of the meta-analysis of the association between HLA-DQ rs9272105 polymorphism and HBV infection outcomes

Comparison	Allele model (A vs. G)			Heterozygous model (AG vs. GG)			Homozygous model (AA vs. GG)			Recessive model (AA vs. AG+GG)			Dominant model (AA+AG vs. GG)		
	OR (95%CI)	*P*_H_	*P*_Z_	OR (95%CI)	*P*_H_	*P*_Z_	OR (95%CI)	*P*_H_	*P*_Z_	OR (95%CI)	*P*_H_	*P*_Z_	OR (95%CI)	*P*_H_	*P*_Z_
HCC vs. CHB	1.31 (1.25-1.37)	0.54	<0.01	1.11 (1.03-1.20)	0.55	0.01	1.70 (1.56-1.86)	0.49	<0.01	1.59 (1.48-1.72)	0.20	<0.01	1.28 (1.19-1.37)	0.69	<0.01

### Evaluation of sensitivity analysis

Sensitivity analysis was performed to assess the effects of an individual study on the overall OR. Meanwhile, the corresponding pooled ORs were not materially altered (Supplementary Figures 1-5).

### Publication bias

Egger’s test was utilized to evaluate the publication bias of the included articles. The data showed that no evidence of publication bias was observed in HLA-DQ region polymorphisms (Supplementary Figures 6-10).

## DISCUSSION

Epidemiologic studies have firmly confirmed that HBV infection plays a pivotal role in the chronic liver disease. To date, immune response has been considered to implicated in HBV clearance and HBV infection [46]. HLAs are members of the major histocompatibility complex (MHC) genes localized on chromosome 6p21, which play important roles in viral infectious diseases [47-49]. In a previous study, Zeng revealed that the proliferative responses of CD4^+^ T cells in patients with acute HBV infection were more severe than those with persistent HBV infection, suggesting MHC class II polymorphisms may affect the susceptibility of subjects to persistent infection [5]. Nowadays, three isotypes of HLA class II molecules have been identified including HLA-DR, HLA-DP, and HLA-DQ, which constitute a heterodimer formed by alpha- and beta-chains on the surface of antigen presenting cells (APCs) including macrophages, dendritic cells (DCs), and B lymphocytes [14]. HLA-DQ proteins, a group of heterodimeric molecules consisted of alpha- and beta-chains encoded by *HLA-DQA1* and *HLA-DQB1* genes [31], were implicated in immune-mediated diseases, including liver diseases and cancer [50, 51]. For example, several SNPs were considered to be associated with persistent HBV infection including HLA-DQA1*0302 [52], -DQB1*0301 [53], and -DQA1*0501 [54].

It is known to all, host genetic factors maybe closely involved in determination of the HBV infection outcome. *HLA-DQ* gene variations, such as *HLA-DQ rs7453920*, *rs2856718*, *rs927210*, *rs9275319* and *rs9275572*, have been regarded to involve in HBV infection or clearance, as well as the disease progression and the development of hepatitis B associated complications (e.g. LC and HCC) [55]. In line with the previous study in Chinese population [28], *HLA-DQ rs7453920* and *rs2856718* SNPs haplotypes showed protective effects in a Japanese population-based study [24]. Hu et al found that *HLA-DQ rs7453920* and *rs2856718* were correlated with increased HBV clearance and decrease of HCC incidence in Han Chinese [28]. Zhang et al study demonstrated that *HLA-DQ rs9275572A* and *rs2856718G* polymorphism were significantly associated with decrease of HBV infection risk and HBV natural clearance. Additionally, *rs9275572A* was also related to the development of cirrhosis and HCC [32]. Interestingly, Al-Qahtani et al results showed that three SNPs (i.e. *rs2856718*, *rs7453920*, and *rs9275572*) of the *HLA-DQ* region contributed to the susceptibility to HBV infection in the Saudi Arabian population [31]. *HLA-DQ rs9275319* was considered as an HBV-HCC susceptible SNP in a GWAS based on the Chinese populations [46], which was different from a previous study [33] in which *rs9275319* variant genotypes were reported to be inversely related to HBV persistence and significantly related to HBV natural clearance [33]. Meanwhile, Li et al revealed that the rs9272105 variant allele was a risk factor for the HCC progression (OR = 1.30) [29]. To date, despite the fact that a large number of publications have focused on the association between HLA-DQ region polymorphisms and HBV infection outcomes, the results are still controversial. In this study, we conducted a meta-analysis to evaluate the relationship between the HLA-DQ region polymorphisms and HBV infection outcomes.

Compared to a single study, meta-analysis can provide sufficient results especially in analyzing unexplained studies [56]. In our previous meta-analysis including 11 case-control studies, we demonstrated that *HLA-DQ rs2856718-G* polymorphism showed protective effects against HBV infection, and *rs2856718-A* was a risk factor for chronic HBV infection [57]. Subsequently, Meta-analysis by Lv et al showed that *rs2856718* and *rs9275572* in HLA-DQ significantly decreased HBV-related HCC in total population, especially in Chinese other than in Saudi Arabian [58]. Whereas, in the analysis stratified by SNPs, only three SNPs (*rs2856718*, *rs7453920*, and *rs9275572*) for HBV infection and/or HBV-related HCC were included, with no study focusing on the *rs9272105* and *rs9275319*. To our best knowledge, this is the first systematic and comprehensive meta-analysis exploring the associations between HLA-DQ region polymorphisms (rs2856718, rs7453920, rs9272105, rs9275319 and rs9275572) and HBV infection outcomes (including HBV infection, CHB, liver cirrhosis, and HBV-related HCC).

Indeed, there are some inherent limitations in this meta-analysis. Firstly, our results were obtained from unadjusted estimates due to lacking of raw data including age, gender, drinking, smoking, lifestyle, as well as environmental factors, which may lead to a confounding bias. Secondly, the number of studies was not large enough for a comprehensive meta-analysis. Thirdly, the gene-gene and of gene-environment interaction has not been evaluated in this study due to absence of original datasets. Finally, a lacking of the original data hampered our further evaluation on the potential interactions between clinical outcomes and viral backgrounds. Therefore, in future, further studies are needed to obtain more reliable results.

In summary, there are really variations between human populations. On this basis, a common SNP allele in a certain geographical or ethnic group may not be commonly observed in another geographical location or population. Our meta-analysis revealed that the HLA-DQ *rs2856718-G* and *rs9275319-G* were significantly associated with decreased risk of HBV infection and HBV natural clearance, but *rs7453920-A* was inconsistent in different populations. Because of the small sample size in Saudi Arabian population in this analysis, our findings need to be validated in future through a population-based study. Logistic regression analysis indicated that *HLA-DQ* allele *rs9275572-A* contributed to the significant increase of HBV infection clearance, and decreased HBV natural clearance. However, *HLA-DQ* alleles *rs2856718-G* and *rs9275572-A* were not associated with development of cirrhosis. The *HLA-DQ* (*rs2856718* and *rs9275572*) polymorphisms were associated with a decreased HBV-related HCC risk in all genetic models, but *HLA-DQ rs9272105* increased the risk of HBV-related HCC, which suggested that CHB patients with *HLA-DQ rs9272105* should be monitored frequently for development of HCC. In addition, no association was observed between *HLA-DQ rs9275319* polymorphism and HBV-related HCC. These findings contribute to the construction of a personalized hepatitis B therapy or prognosis in the near future.

## MATERIALS AND METHODS

### Literature search strategy

Literature search was performed from PubMed, EMBASE, China National Knowledge Infrastructure (CNKI) and Chinese WanFang databases, using the following keywords: “HLA-DQ”, “hepatitis B virus” or “HBV”, “HBV clearance” or “HBV natural clearance” or “NC”, “chronic hepatitis B” or “CHB”, “liver cirrhosis” or “LC” or “cirrhosis”, “Hepatocellular carcinoma” or “HCC” or “liver cancer”, “polymorphism” or “Single Nucleotide Polymorphism” or “SNP”, and “rs2856718” or “rs7453920” or “rs9272105” or “rs9275319” or “rs9275572”. Only the literatures published before June 21, 2017 were included, and were reviewed by two independent investigators (Tao Xu and Anyou Zhu). The search focused only on full articles for the meta-analysis. No language restriction was applied in the search process.

### Inclusion and exclusion criteria

Eligible studies should meet the inclusion criteria as follows: (1) case-control studies; (2) studies with sufficient data for the estimation of an odds ratio (OR) with 95% confidence interval (CI); (3) studies reporting the genotype frequencies; (4) in cases of the same group of patients reported in multiple studies, only the most informative study was used to avoid duplication. The exclusion criteria were as follows: (1) duplicate data; (2) review articles; (3) case-only studies; (4) lacking of genotype frequency data; (5) with no full text available.

### Quality assessment

Newcastle-Ottawa Scale (NOS) was applied to assess the quality of each included study [27]. The quality of studies was scored based on the following criteria: selection of cases, comparability of populations, and ascertainment of exposure to risks. Studies with a score of ≥ 6 were considered to be of high quality. In cases of any disagreement on the assigned grade, studies were fully reassessed until a consensus was achieved.

### Data extraction

For the data extraction, the following data were independently extracted from the eligible studies: first author, publication date, ethnicity, genotyping method, cases stratified as HBV-related HCC, LC, and/or CHB; controls including the healthy controls and HBV clearance controls, total numbers of cases and controls. Two investigators (Tao Xu and Anyou Zhu) checked the data extraction results, and an open discussion or consultation was held in the presence of any disagreements.

### Statistical analysis

SNP data were divided into four groups: HBV infection vs. healthy controls; HBV infection vs. NC; LC vs. CHB; HCC vs. (CHB and/or LC). The significance for five genetic models (allele model, heterozygous model, homozygous model, recessive model, and dominant model) was evaluated for each study, respectively. Statistical analysis was performed using STATA software (version 12.0; Stata Corporation, College Station, Texas, USA). Hardy-Weinberg equilibrium test (HWE) was evaluated for controls in each study by using the χ^2^-test, and P <0.05 was considered as departure from HWE. All the associations were estimated by ORs and 95% CIs. The significance of the pooled ORs was determined by Z-test and P <0.05 was considered statistically significant. Potential heterogeneity was evaluated using a χ^2^-based Q-test. *P*_H_ ≥ 0.05 indicated a lack of heterogeneity among studies, and a fixed-effect model was used. Otherwise, a random-effects model was applied. Sensitivity analysis was performed by omitting each study in turn to determine the effects on the test of heterogeneity. Publication bias of literatures was assessed by Begg’s funnel plot.

## SUPPLEMENTARY MATERIALS FIGURES


