# Supplementary material for: Quantitative assessment of HLA-DQ gene polymorphisms with the development of hepatitis B virus infection, clearance, liver cirrhosis, and hepatocellular carcinoma
Source: Oncotarget. 2017 Dec 5;9(1):96–109. doi: 10.18632/oncotarget.22941 (PMC5787527; doi:10.18632/oncotarget.22941)
Supplement: Supplementary file 1 [file oncotarget-09-96-s001.pdf]

Quantitative assessment of HLA-DQ gene polymorphisms with the development of hepatitis B virus infection, clearance, liver cirrhosis, and hepatocellular carcinoma

SUPPLEMENTARY MATERIALS

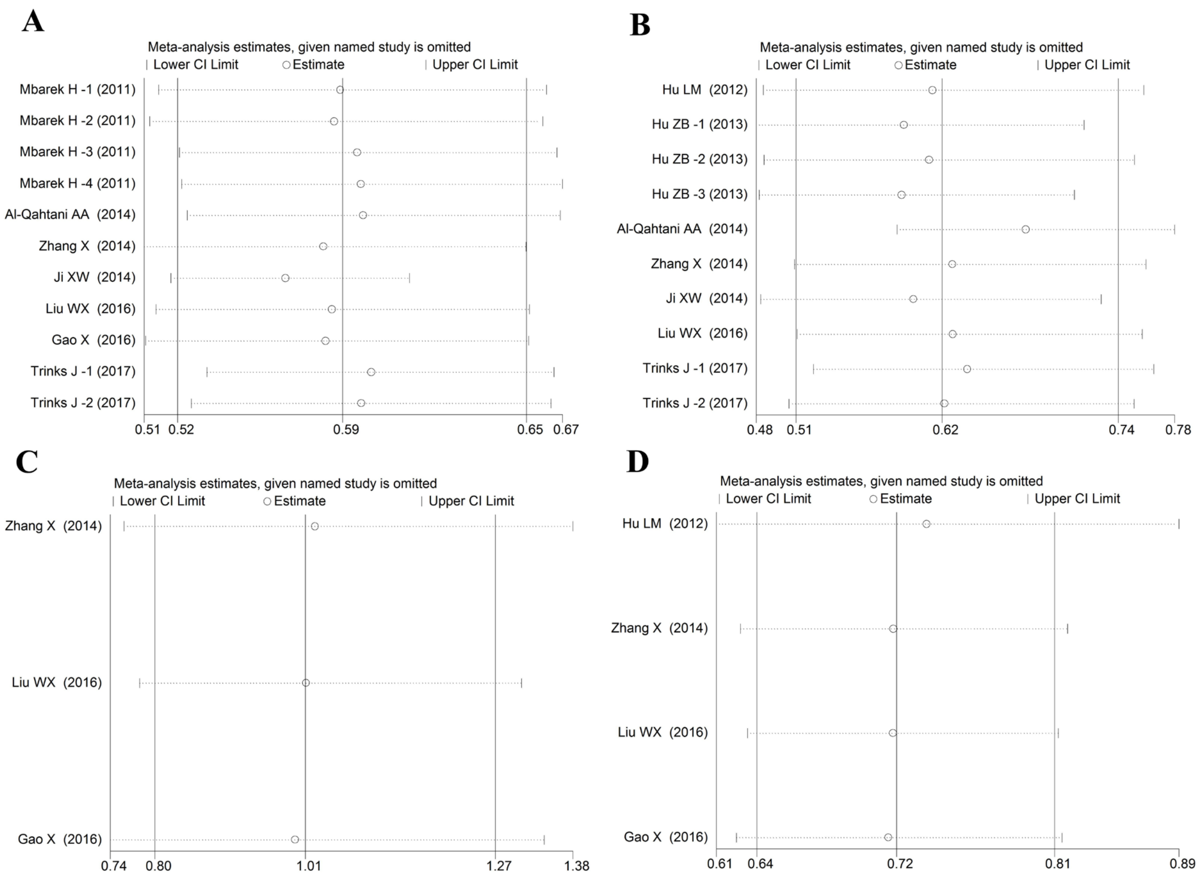

Supplementary Figure 1: Sensitivity analysis of the pooled ORs and 95%CIs for HLA-DQ rs2856718 polymorphism. **A**. HBV infection vs. Control (AA vs. AG+GG); **B**. HBV infection vs. NC (AA vs. AG+GG); **C**. LC vs. CHB (AA vs. AG+GG); D. HCC vs. LC+CHB (AA vs. AG+GG)

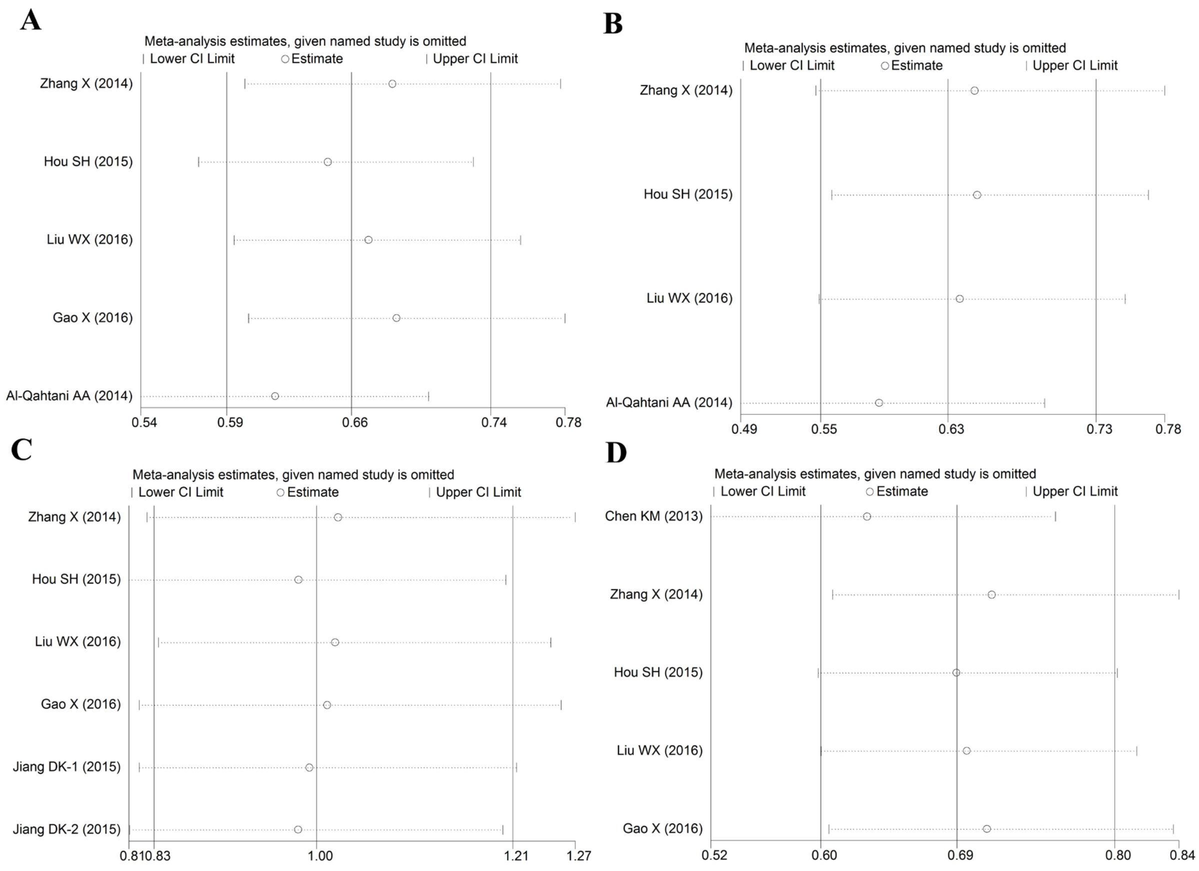

Supplementary Figure 2: Sensitivity analysis of the pooled ORs and 95%CIs for HLA-DQ rs9275572 polymorphism. **A**. HBV infection vs. Control (AA vs. AG+GG); **B**. HBV infection vs. NC (AA vs. AG+GG); **C**. LC vs. CHB (AA vs. AG+GG); **D**. HCC vs. LC+CHB (AA vs. AG+GG)

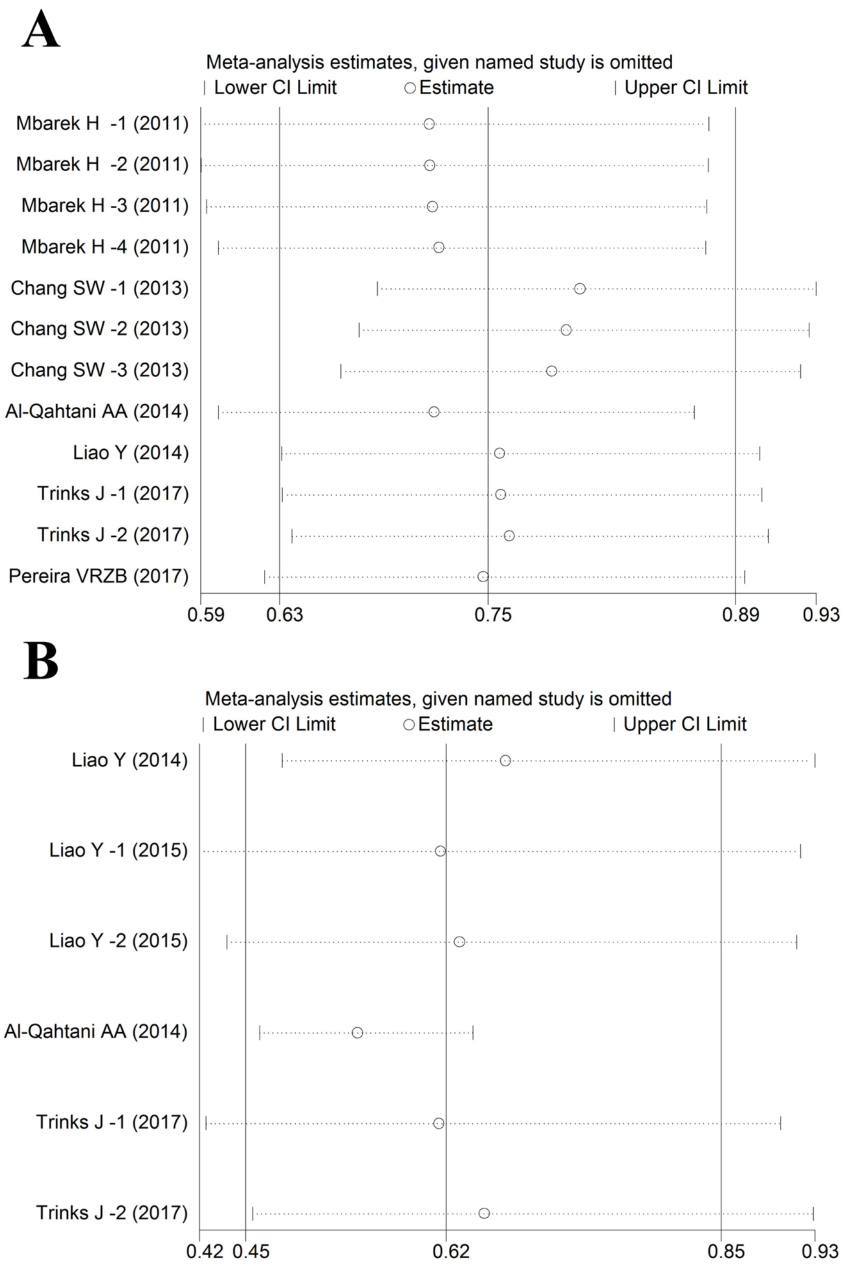

Supplementary Figure 3: Sensitivity analysis of the pooled ORs and 95%CIs for HLA-DQ rs7453920 polymorphism.
A. HBV infection vs. Control (AA vs. AG+GG); **B**. HBV infection vs. NC (AA vs. AG+GG)

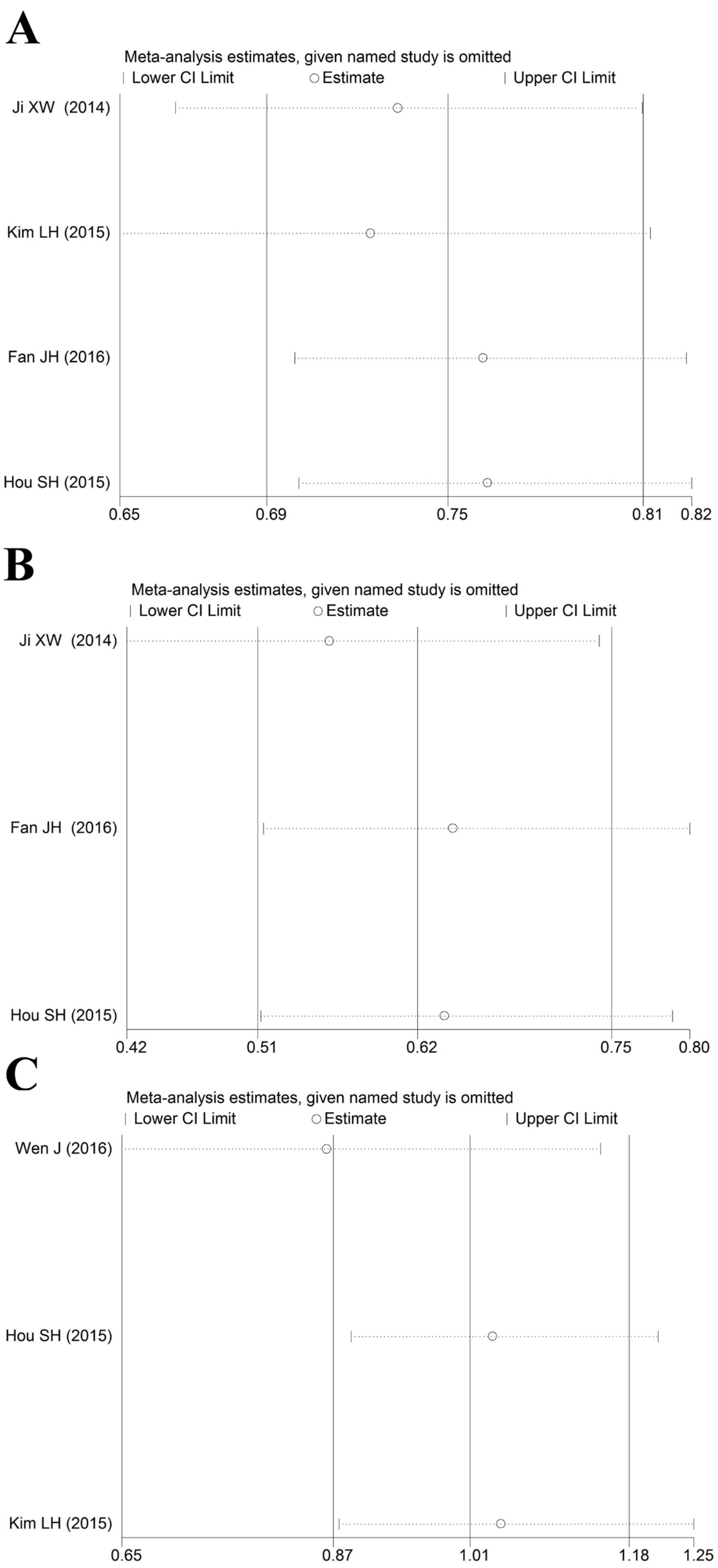

Supplementary Figure 4: Sensitivity analysis of the pooled ORs and 95%CIs for HLA-DQ rs9275319 polymorphism. **A**. HBV infection vs. Control (AA vs. AG+GG); **B**. HBV infection vs. NC (AA vs. AG+GG); **C**. HCC vs. LC+CHB (AA vs. AG+GG)

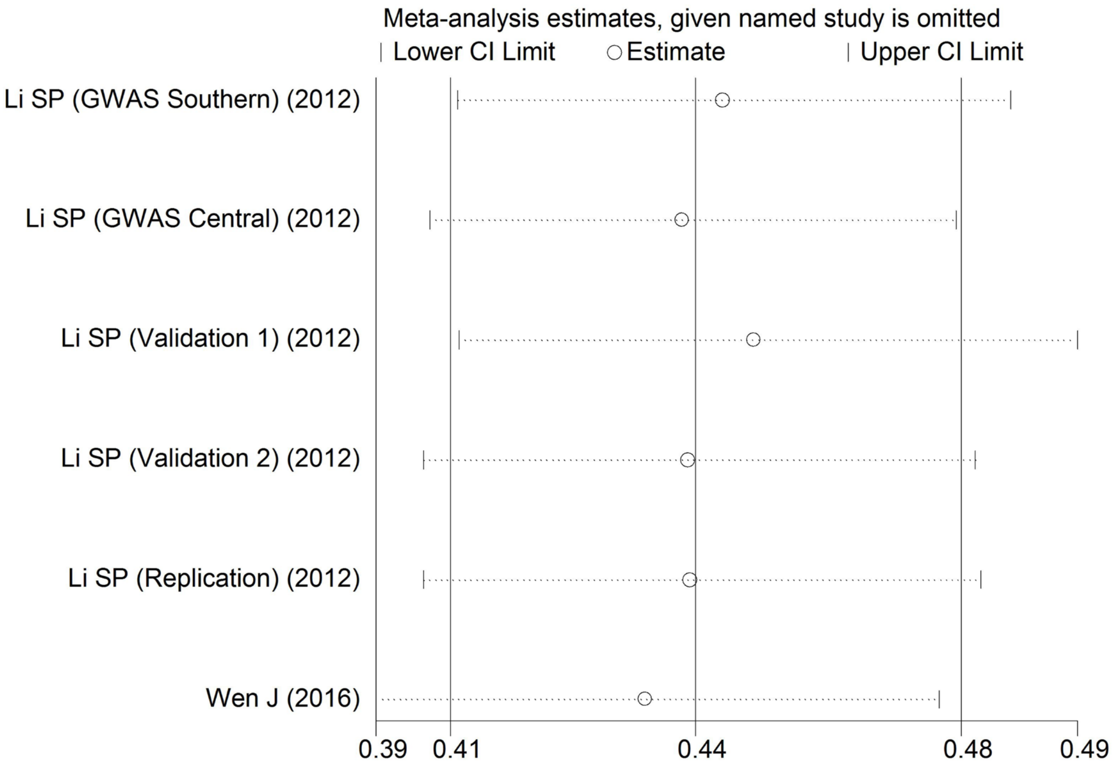

Supplementary Figure 5: Sensitivity analysis of the pooled ORs and 95%CIs for HLA-DQ rs9272105 polymorphism. HCC vs. LC+CHB (AA vs. AG+GG)

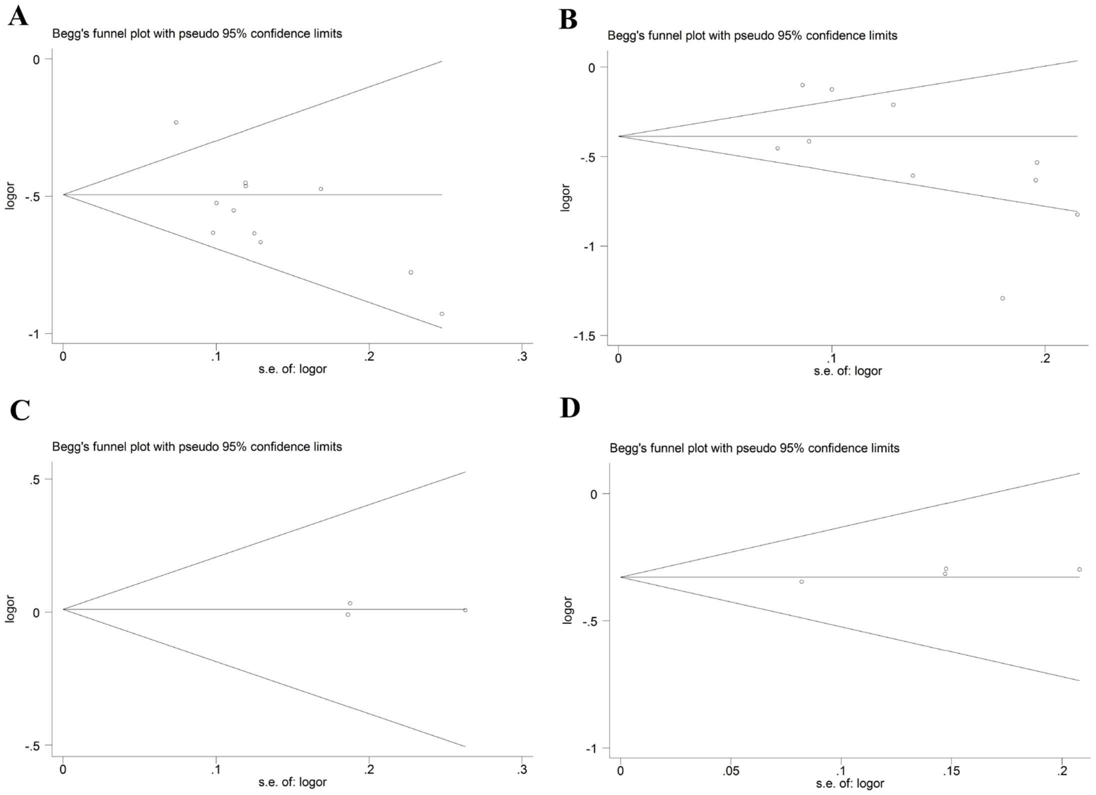

Supplementary Figure 6: Funnel plot analysis of the association of HLA-DQ rs2856718 polymorphism with HBV infection outcomes. **A**. HBV infection vs. Control (AA vs. AG+GG); **B**. HBV infection vs. NC (AA vs. AG+GG); **C**. LC vs. CHB (AA vs. AG+GG); **D**. HCC vs. LC+CHB (AA vs. AG+GG)

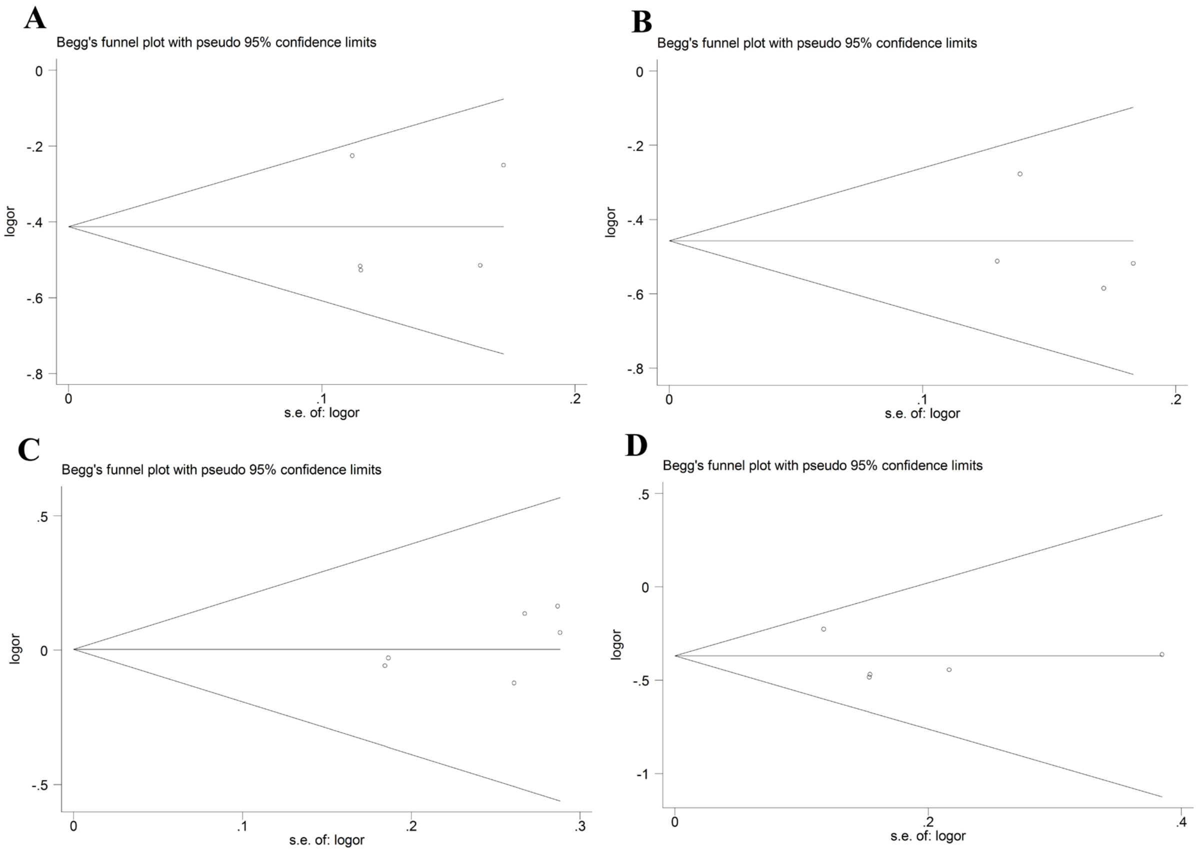

Supplementary Figure 7: Funnel plot analysis of the association of HLA-DQ rs9275572 polymorphism with HBV infection outcomes. **A**. HBV infection vs. Control (AA vs. AG+GG); **B**. HBV infection vs. NC (AA vs. AG+GG); **C**. LC vs. CHB (AA vs. AG+GG); D. HCC vs. LC+CHB (AA vs. AG+GG)

A

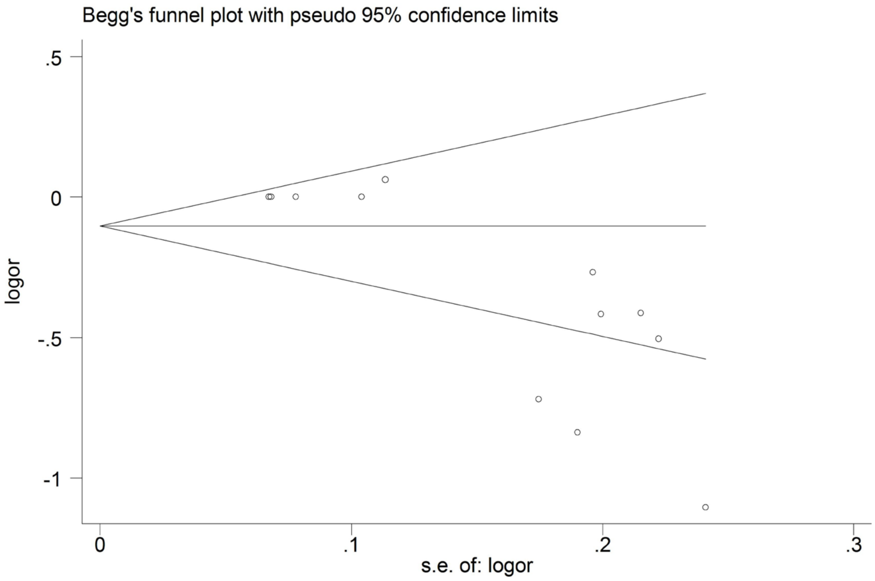

B

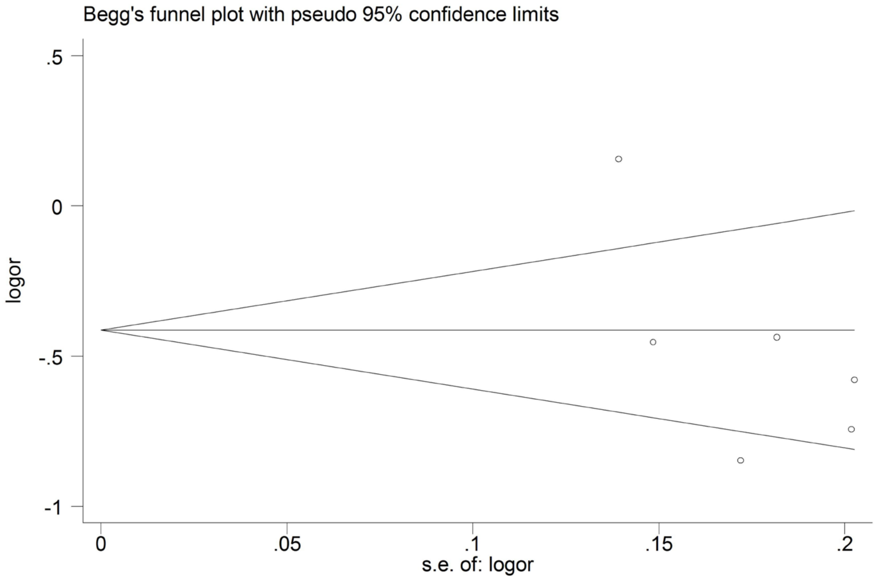

Supplementary Figure 8: Funnel plot analysis of the association of HLA-DQ rs7453920 polymorphism with HBV infection outcomes. A. HBV infection vs. Control (AA vs. AG+GG); **B**. HBV infection vs. NC (AA vs. AG+GG)

A

B

C

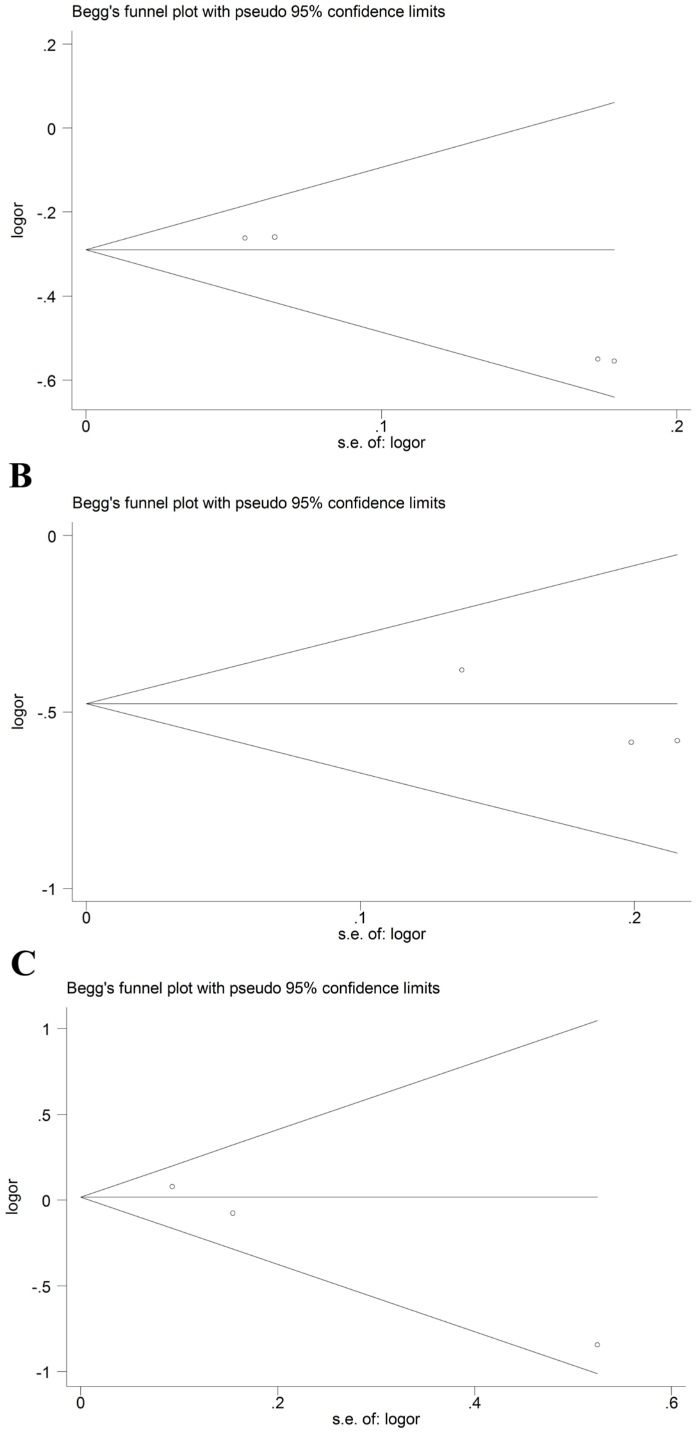

Supplementary Figure 9: Funnel plot analysis of the association of HLA-DQ rs9275319 polymorphism with HBV infection outcomes. **A**. HBV infection vs. Control (AA vs. AG+GG); **B**. HBV infection vs. NC (AA vs. AG+GG); **C**. HCC vs. LC+CHB (AA vs. AG+GG)

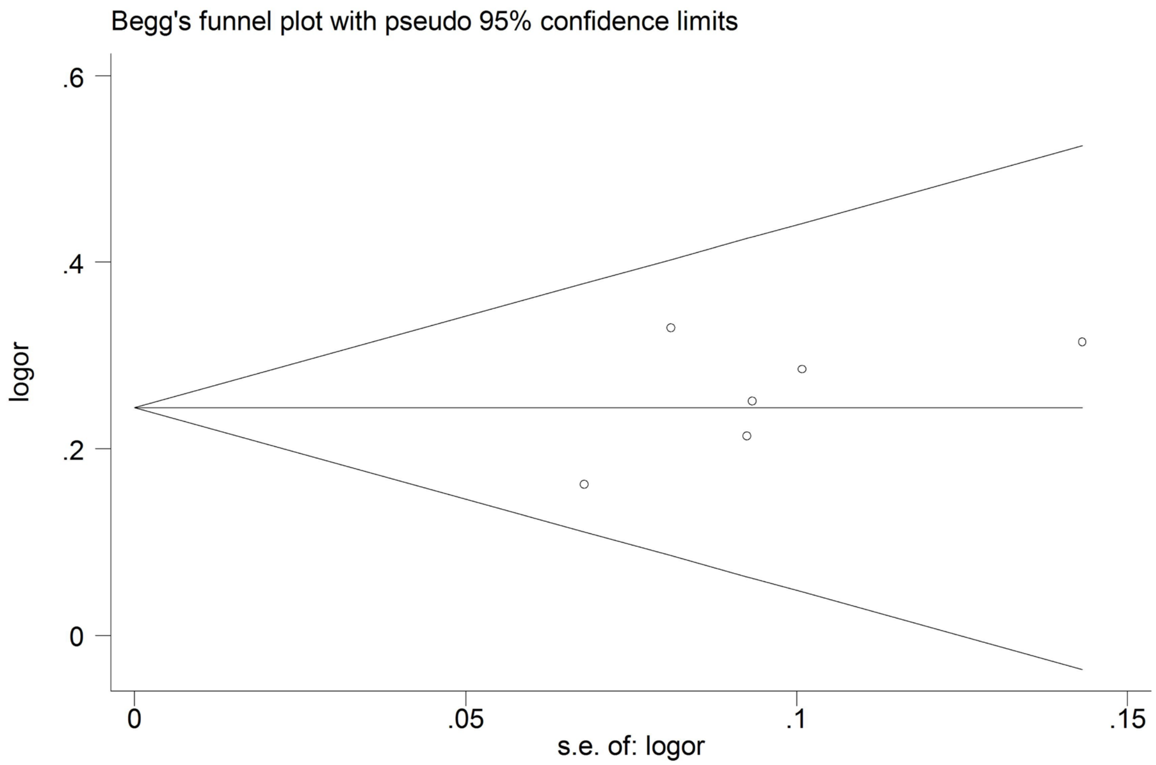

Supplementary Figure 10: Funnel plot analysis of the association of HLA-DQ rs9272105 polymorphism with HBV infection outcomes. HCC vs. LC+CHB (AA vs. AG+GG)
